# Genetic and Clinical Characterization of TANGO2 Deficiency Disorder: Insights from the Italian Multicentre Cohort

**DOI:** 10.3390/ijms27104389

**Published:** 2026-05-14

**Authors:** Emanuela Claudia Turco, Giulia Pisanò, Laura Caiazza, Silvia Carestiato, Benedetta Piccolo, Simona Fecarotta, Francesca Pochiero, Federica Ricci, Alfredo Brusco, Giovanni Battista Ferrero, Susanna Esposito, Carlo Fusco, Maria Carmela Pera

**Affiliations:** 1 Child Neuropsychiatric Unit, Maternal and Child Health Department, Parma University-Hospital, 43126 Parma, Italy; bpiccolo@ao.pr.it; 2 Child Neurology and Psychiatry Unit, Department of Pediatrics, Presidio Ospedaliero Santa Maria Nuova, AUSL-IRCCS di Reggio Emilia, 42123 Reggio Emilia, Italy; giuliapisan@gmail.com (G.P.); carlo.fusco@ausl.re.it (C.F.); 3 Department of Biomedical, Metabolic and Neural Sciences, University of Modena and Reggio Emilia, 41125 Modena, Italy; laura.caiazza96@gmail.com; 4 Department of Neuroscience “Rita Levi-Montalcini”, University of Turin, 10124 Torino, Italy; silvia.carestiato@unito.it (S.C.); alfredo.brusco@unito.it (A.B.); 5 Section of Pediatrics, Metabolic Diseases Unit, Department of Translational Medical Science, Federico II University of Naples, 80131 Naples, Italy; simona.fecarotta@gmail.com; 6 Metabolic Disease Unit, Neuroscience Department, Meyer Children Hospital, 50139 Florence, Italy; francesca.pochiero@meyer.it; 7 Neuromuscular Center, AOU Città della Salute e della Scienza, University of Turin, 10126 Torino, Italy; federica.ricci@unito.it; 8 Medical Genetics Unit, Città della Salute e della Scienza University Hospital, 10126 Turin, Italy; 9 Department of Clinical and Biological Sciences, University of Turin, 10043 Orbassano, Italy; giovannibattista.ferrero@unito.it; 10 Pediatric Clinic, Department of Medicine and Surgery, University of Parma, 43126 Parma, Italy; susannamariaroberta.esposito@unipr.it; 11 Child Neuropsychiatry Unit, Department of Medicine and Surgery, University of Parma, 43126 Parma, Italy; mariacarmela.pera@unipr.it

**Keywords:** TANGO2, metabolic crisis, neurodevelopmental delay, rare genetic disease, genotype–phenotype correlation

## Abstract

TANGO2-deficiency disorder (TDD) is a rare autosomal recessive condition characterised by neurodevelopmental delay, TANGO2 spells, life-threatening metabolic crises, and cardiac arrhythmias. Genotype–phenotype correlations remain poorly defined and the neurobehavioural profile of affected individuals is largely unexplored. We conducted a retrospective multicentre study of five Italian patients with genetically confirmed TDD, identified between June 2023 and May 2025. Clinical, neurophysiological, neuroimaging, genetic, and neurodevelopmental data were collected. Adaptive functioning, cognitive ability, and behavioural profiles were assessed using standardised instruments. All five patients carried biallelic *TANGO2* mutations, including two previously unreported variants. Clinical severity ranged from an asymptomatic individual under preventive therapy to a fatal early-onset metabolic crisis. Marked intrafamilial variability was observed in two siblings sharing the same genotype. Systematic neurodevelopmental assessment revealed a spectrum of cognitive and adaptive outcomes, with attentional difficulties identified as a recurrent feature. No metabolic crises or TANGO2 spells were documented following initiation of B-vitamin and cofactor supplementation in surviving patients. This cohort expands the mutational and phenotypic spectrum of TDD and highlights the diagnostic value of *TANGO2* testing in patients with neurodevelopmental delay or paroxysmal neurological episodes, even in the absence of metabolic crises. Early supplementation therapy may contribute to clinical stability, though prospective controlled studies are needed.

## 1. Introduction

TANGO2-deficiency disorder (TDD; OMIM #616878) is a rare autosomal recessive condition first described in 2016, caused by biallelic pathogenic variants in the *TANGO2* gene [1,2,3]. To date, over 100 individuals have been diagnosed worldwide; given an estimated prevalence of approximately 1:1,000,000, the true number of affected individuals may be substantially higher [2,3,4].

The precise function of the TANGO2 protein is still being elucidated. Recent evidence identifies it as an acyl-CoA binding protein, suggesting a role in shuttling acyl-CoA from the cytoplasm to the mitochondrial lumen to support β-oxidation and lipid metabolism [5]. Converging lipidomic and functional studies further implicate TANGO2 in phospholipid homeostasis, vesicular trafficking, and mitochondrial dynamics [6,7,8], with lipid imbalance emerging as a central pathogenic mechanism [7,8]. The clinical consequences of TANGO2 dysfunction are correspondingly broad, and phenotypic expression varies considerably even among individuals sharing identical biallelic variants, suggesting a role for genetic modifiers and environmental factors [4].

Neurological involvement is the most consistent feature of TDD, encompassing variable degrees of cognitive impairment, gross and fine motor dysfunction, and speech delay [4]. Epilepsy occurs in approximately 40–50% of patients, with drug-resistant seizures in roughly one third [4,9]. Nearly two thirds of individuals experience TANGO2-related paroxysmal neurological episodes—termed TANGO2 spells—typically emerging between one and three years of age [4]. These episodes are characterised by abrupt worsening of baseline neurological symptoms, including hypotonia, ataxia, dysarthria, drooling, and altered alertness and are commonly precipitated by fasting, febrile illness, physical exertion, or thermal stress [4]. Although self-limiting, TANGO2 spells may herald a metabolic crisis. Such crises affect approximately two thirds of TDD patients [2,3,10,11] and represent the principal cause of morbidity and mortality in this disorder [12]. They are biochemically characterised by hypoglycaemia, hyperammonaemia, lactic acidosis, and rhabdomyolysis, and frequently progress to life-threatening cardiac involvement, including markedly prolonged QTc interval, transient type 1 Brugada pattern, ventricular arrhythmias, and cardiomyopathy [4,12].

Early supplementation with B-complex vitamins—particularly pantothenic acid (vitamin B5), a precursor of coenzyme A—has shown efficacy in reducing the frequency and severity of metabolic crises in a subset of patients [4,13]. The underlying mechanism likely involves a combination of antioxidant effects, enhanced mitochondrial function, and support of de novo lipid biosynthesis [13,14]. More recently, vitamin B5 monotherapy has been reported to improve clinical outcomes in individual cases [15], further supporting its therapeutic relevance.

Despite growing recognition of TDD, the phenotypic spectrum and natural history of the disorder remain incompletely characterised, and genotype–phenotype correlations are poorly defined [16]. Here we report a two novel *TANGO2* variants. We describe the clinical, neurophysiological, neuroimaging and neurodevelopmental features of this cohort and provide longitudinal follow-up data that expand our understanding of phenotypic variability and support the role of early intervention in improving neurodevelopmental trajectories.

## 2. Results

### 2.1. Clinical Findings

**Figure 1:**
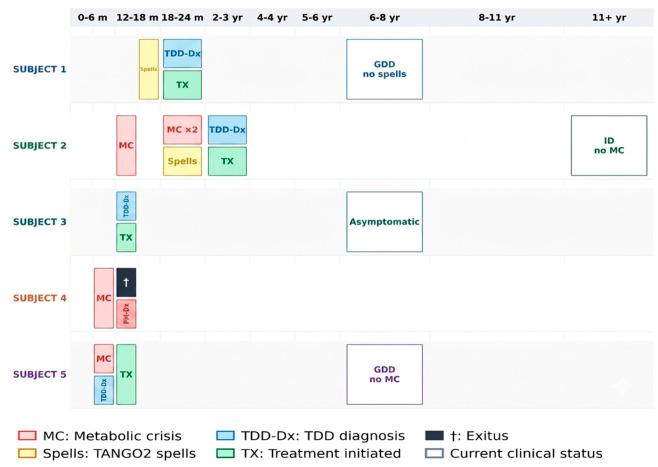
Clinical timelines of five patients with TANGO2–deficiency disorder. Each row represents one patient. Coloured boxes indicate clinical events and interventions within the corresponding age interval. MC: metabolic crisis; Spells: TANGO2–related paroxysmal neurological episodes; TDD–Dx: confirmed diagnosis of TANGO2–deficiency disorder; TX: initiation of vitamin and cofactor supplementation; PM–Dx: post-mortem diagnosis; †: deceased. Outlined boxes without fill indicate current clinical status at last follow-up. GDD: global developmental delay; ID: intellectual disability; MC × 2: two consecutive metabolic crises.

**Figure 2:**
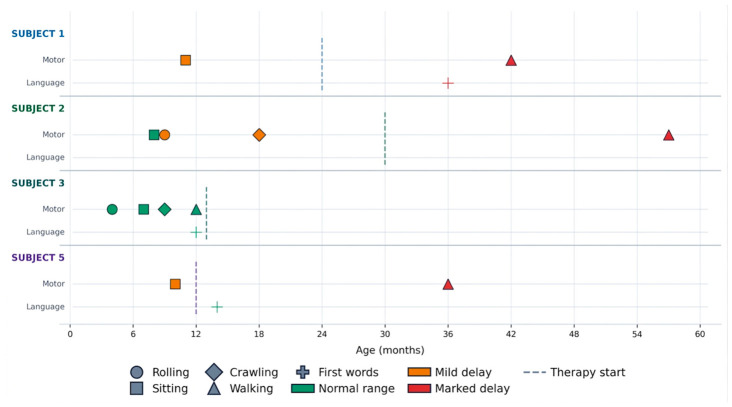
Developmental milestone timeline in patients with TANGO2–deficiency disorder. Motor and language milestones are plotted by age for Subjects 1–3 and 5. Symbol shape indicates milestone type (○ rolling, □ sitting, ◇ crawling, △ walking, and + first words); colour indicates developmental status (green: within normal range; yellow: mild delay; red: marked delay ≥ 2× upper limit). The dashed vertical line marks therapy initiation. Subject 4 excluded (deceased at 13 months).

**Figure 3:**
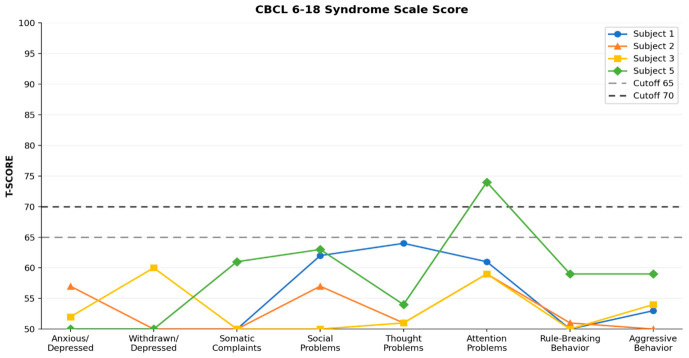
CBCL 6–8 Syndrome Scale T-scores for Subjects 1, 2, 3, and 5. Dashed lines indicate the borderline clinical cutoff (T = 65) and the clinical cutoff (T = 70). Subject 4 is not included as the patient was deceased prior to assessment. Only the Attention Problems scale exceeded the clinical cutoff, observed exclusively in Subject 5.

**Figure 4:**
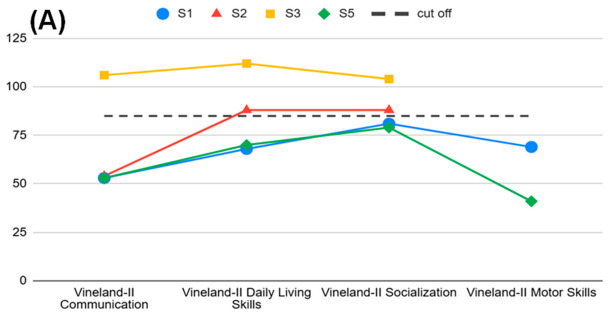

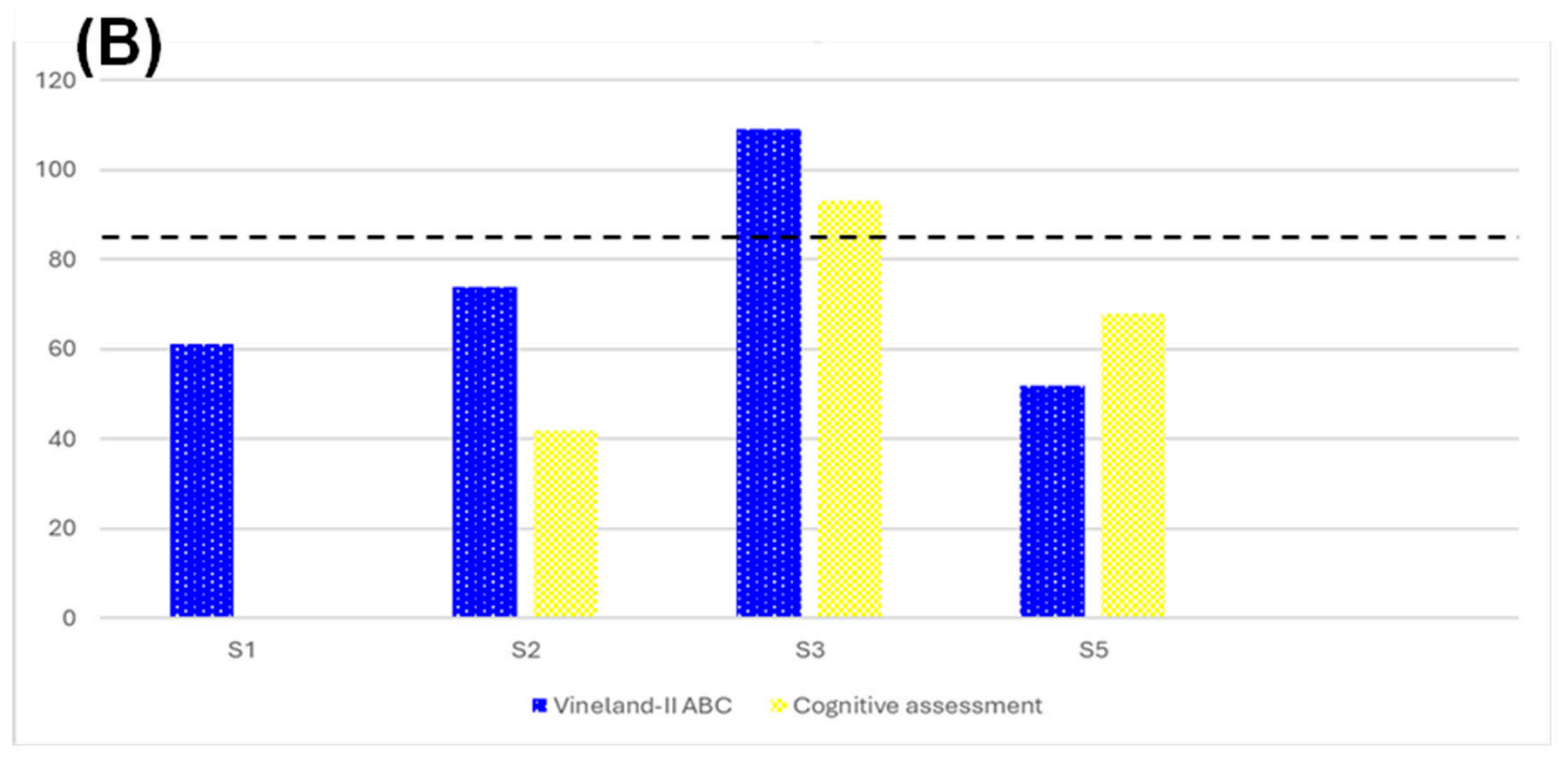
(**A**) Vineland–II domain standard scores for Subjects 1, 2, 3, and 5. Dashed line indicates the normative cutoff (SS = 85). (**B**) Comparison between Vineland-II Adaptive Behaviour Composite (ABC) and intellectual quotient (IQ) scores across subjects. For Subject 2, IQ was assessed with the WISC–IV (FSIQ 42); for Subject 3, with the WISC–IV (FSIQ 93); for Subject 5, with the Leiter–3 (nonverbal IQ 68). Subject 1 was not testable by direct cognitive assessment; Subject 4 is not included (deceased prior to assessment). The dashed line indicates the normative cutoff (SS/IQ = 85).

**Figure 5:**
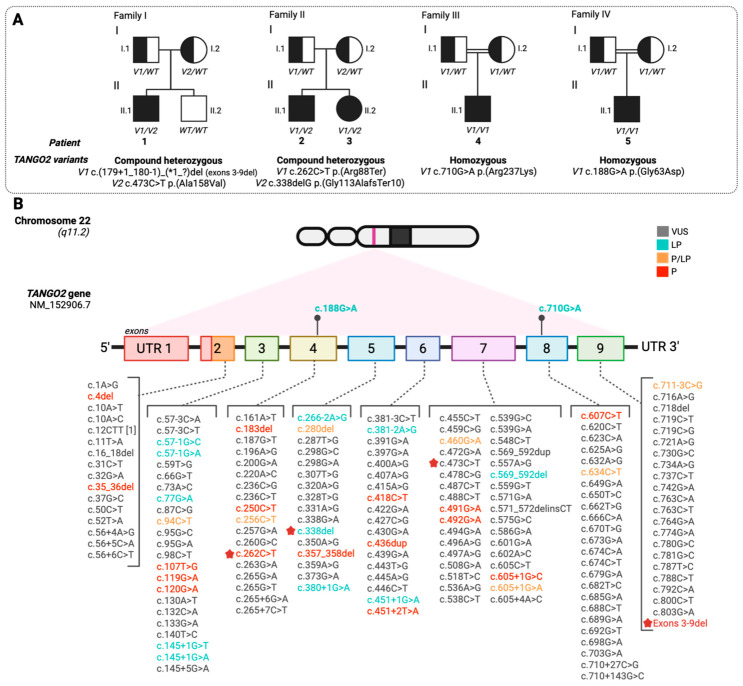
Segregation pattern in our cohort and *TANGO2* gene illustration with variants ranging from uncertain significance to most deleterious from this study and previously reported patients. (**A**) Pedigrees of the four families included in this study, showing the segregation of *TANGO2* variants in affected individuals. Each pedigree displays the affected patients, their genotypes, and the inheritance patterns of the identified variants, emphasising familial transmission. (**B**) Illustration of reported *TANGO2* variants sourced from the ClinVar database, based on transcript NM_152906.7. A total of 170 variants are classified according to their clinical significance, following the ACMG criteria: variant of uncertain significance (VUS, gray), likely pathogenic (LP, green), pathogenic/likely pathogenic (P/LP, orange), and pathogenic (P, red). Variants identified in our patients that have been previously reported are labelled with a red star (exons 3–9del; c.262C>T; c.338del; c.473C>T). Novel variants are highlighted in bold and positioned above the corresponding exons using a lollipop bar (c.188G>A; c.710G>A). This visualisation provides an overview of the mutational landscape of the *TANGO2* gene, aiding in the interpretation of genetic alterations associated with disease. Asterisk indicates variants identified in our patients that have been previously reported.

Table 1 individual clinical timelines in Figure 1, developmental milestones in Figure 2, behavioural profiles in Figure 3, adaptive and cognitive profiles in Figure 4, and genetic variant characterization in Table 2 and Figure 5.

Subject 1. A 6-year-old male presented with global psychomotor delay (sitting at 11 months, first words at 36 months, and independent walking at 42 months). From 23 months of age, he developed recurrent paroxysmal neurological episodes fulfilling criteria for TANGO2 spells, characterised by abrupt onset of axial hypotonia, involuntary dystonic posturing, head tilt, emesis and behavioural irritability with preserved but reduced alertness. Spells occurred multiple times per month, were precipitated by febrile illness and insufficient caloric intake, lasted several hours, and resolved spontaneously with prolonged fatigue; no consistent circadian pattern was identified. No acute pharmacological intervention was required, and metabolic parameters obtained during and after episodes remained within normal ranges. Interictal electroencephalogram (EEG) showed bitemporal background slowing with superimposed fast rhythms and occasional sharp waves; no ictal discharges were recorded during paroxysmal episodes, supporting their non-epileptic nature. Brain MRI revealed bilateral temporopolar arachnoid cysts with ventriculomegaly, considered incidental findings. Cardiac evaluation including electrocardiogram (ECG) with QTc measurement was normal. Baseline metabolic workup—including creatine phosphokinase (CPK), liver enzymes, lactate, ammonia, plasma amino acids, acylcarnitine profile, urine organic acids, very-long-chain fatty acids, and CSF neurotransmitters—was within normal limits. Family-based exome sequencing identified compound heterozygous pathogenic variants in *TANGO2*: a paternal multiexonic deletion (exons 3–9) and a maternal missense variant (NM_152906.7: c.473C>T; p.Ala158Val), confirming the diagnosis of TDD (Table 2, Figure 5). Treatment with CoQ10, vitamin A, B-complex (B1, B5, B6, and B9), vitamin C, vitamin D3, L-carnitine, probiotics and melatonin was initiated at 24 months of age (see Table 1). Following initiation of supplementation therapy, complete resolution of spells was observed. Formal standardised testing could not be completed due to behavioural challenges and limited engagement. Based on caregiver-reported Vineland-II and clinical observation, significant adaptive impairment was documented across communication, daily living skills, and motor domains, with features of oro-verbal dyspraxia and fine motor impairment and relative strengths in domestic activities and gross motor functioning. Behavioural assessment using the CBCL indicated borderline scores on the Thought Problems and Attention Problems scales, with all remaining scales within normal limits. This patient has been previously reported [18].

Subject 2. An 11-year-old male, older sibling of Subject 3, first presented at 13 months with a severe metabolic crisis featuring rhabdomyolysis, QTc prolongation, and cardiomyopathy with mildly reduced systolic ejection fraction. Hypothyroidism was identified as a pre-existing comorbidity at the time of initial evaluation. Two subsequent metabolic crises at 27 and 28 months were complicated by markedly elevated CK levels (peak 226,844 IU/L) and cardiogenic shock requiring intensive care. From 2.5 years of age, recurrent paroxysmal episodes consistent with TANGO2 spells were documented, typically preceding the metabolic crises and characterised by ataxia, sialorrhea, lethargy, hypotonia, and generalised weakness, precipitated by febrile illness and reduced oral intake. Interictal EEG was unremarkable; brain MRI was not performed. Exome sequencing identified compound heterozygous pathogenic variants in *TANGO2*: a paternal nonsense variant and a maternal frameshift variant (NM_152906.7: c.262C>T; p.Arg88Ter and c.338delG; p.Gly113AlafsTer10) (Table 2, Figure 5). At 20 months, Bayley-III assessment yielded a composite score of 55 (6-month age equivalent), documenting severe global developmental delay and language impairment. At current age, the clinical picture is characterised by intellectual disability (WISC-IV Full Scale Intelligence Quotient—FSIQ 42), muscle weakness and hypotonia with a wide-based independent gait; complex visual impairment is also present. Despite persistent deficits, a modest improvement in the overall developmental trajectory has been observed over time. Behavioural assessment using the CBCL revealed a borderline score on the Withdrawn/Depressed scale, with all remaining scales within normal limits. Caregiver-reported Vineland-II indicated adequate adaptive functioning in the Daily Living Skills and Socialization domains, with low performance in Communication. No further metabolic crises have been documented since initiation of the full supplementation regimen with B-complex (B1, B2, B5, B6, B9, B12), vitamins A, C, D, ubiquinol, ubidecarenone, L-carnitine and low-fat high-carbohydrate diet at age of 30 months (Table 1). This patient has been previously reported [19,20].

Subject 3. A 9-year-old female, younger sibling of Subject 2, was diagnosed at 13 months via predictive genetic testing following her brother’s diagnosis, and preventive treatment with B-complex with vitamin C, pantothenic acid, ubidecarenone, L-carnitine and low-fat high-carbohydrate diet was initiated promptly. Unlike her sibling, she has achieved all developmental milestones within normal limits and demonstrates age-appropriate cognitive functioning (WISC-IV FSIQ 93). She presents with only mild learning difficulties in reading, writing and logical-mathematical skills, for which a personalised learning plan has been implemented. She has remained free of TANGO2 spells and metabolic crises to date. EEG and brain MRI were unremarkable. Genetic testing revealed the same compound heterozygous variants as her brother. Caregiver-reported Vineland-II indicated adequate adaptive functioning across all domains. Behavioural assessment using the CBCL showed all syndrome scales within normal limits. This case has been previously reported [19,20].

Subject 4. A male infant born to consanguineous parents presented with failure to thrive and developmental regression from 6 months of age, predating any documented metabolic decompensation. At 9 months, he experienced a severe metabolic crisis characterised by seizures and markedly elevated CK levels. He died at 13 months following massive rhabdomyolysis and cardiac arrest. Interictal EEG showed posterior high-voltage activity. Brain MRI performed during the acute metabolic crisis revealed increased T2 signal in the brainstem and basal ganglia. Post-mortem family-based exome sequencing identified a homozygous missense variant in *TANGO2* (NM_152906.7: c.710G>A; p.Arg237Lys), confirming the diagnosis of TDD (Table 2, Figure 5).

Subject 5. A 6-year-old female, born to second-degree cousins, presented at 9 months with hypotonia, somnolence and anorexia. A severe metabolic crisis at presentation revealed rhabdomyolysis (CK 34,400 IU/L), hyperammonaemia (147 µmol/L), elevated lactate, elevated transaminases and troponin I, prolonged QTc, and an abnormal acylcarnitine profile. Thyroid function and CK levels remained within normal limits on subsequent evaluations. Brain MRI was not performed due to parental refusal of sedation. Initial EEG showed sleep-activated posterior and frontal spikes; a follow-up recording demonstrated diffuse low-voltage theta activity with well-organised sleep architecture, representing improvement from prior studies. No TANGO2 spells were ever documented. Exome sequencing identified a homozygous missense variant (NM_152906.7:c.188G>A; p.Gly63Asp), confirming TDD (Table 2, Figure 5). Supplementation therapy was initiated at 1 year of age (Table 1), with no further rhabdomyolysis episodes over five years of follow-up. Current treatment includes propranolol, B-complex (thiamine, riboflavin, pyridoxine, and pantothenic acid), CoQ10, vitamin C, and N-acetylcysteine. Formal neuropsychological evaluation conducted approximately 18 months after pantothenic acid initiation revealed a nonverbal IQ of 68 (2nd percentile, Leiter-3). Caregiver-reported Vineland-II showed a non-unitary adaptive profile due to significant inter-domain scatter, with low performance in Communication and Motor Skills and moderately low performance in Socialisation and Daily Living Skills. To assess the attentive and hyperkinetic component, the Conners’ Parent Rating Scale—Revised: Long Form (CPRS-R:L) was administered to caregivers; the results indicated a markedly atypical symptom range (T-score >70) on the Cognitive Problems/Inattention, ADHD Index, and CGI Restless-Impulsive subscales. Behavioural assessment using the CBCL identified a clinically significant score on the Attention Problems scale (above the clinical cutoff), with borderline scores on Social Problems and Rule-Breaking Behaviour, and all remaining scales within normal limits. Given clinical suspicion for autism spectrum disorder, a formal evaluation was conducted using the Childhood Autism Rating Scale (CARS) and the Short Sensory Profile; both were negative for relevant symptomatology.

### 2.2. Molecular Characterisation of TANGO2 Variants

The *TANGO2* gene (22q11.21) encodes a multifunctional protein involved in mitochondrial function, lipid metabolism, and vesicular trafficking. According to gnomAD, it displays a pLI score of 0 and a missense Z-score of 0.6, suggesting that heterozygous missense changes are tolerated, while biallelic loss-of-function (LoF) variants are consistent with disease expression, in line with its autosomal recessive inheritance. All five patients harboured biallelic variants in transcript NM_152906.7, including two novel variants not previously reported in ClinVar or Decipher. The identified alleles included frameshift, nonsense, missense, and multiexonic deletion variants, with two affecting potential splicing sites. Pathogenicity was assessed using multiple in silico tools and genomic resources, including: Franklin, ClinVar, MutationTaster, SpliceAI, Pangolin, gnomAD, and the UCSC Genome Browser; CADD scores above 20 for all variants, supporting high deleterious potential; phyloP100way conservation scores ranging from 0.9 to 8.5; SpliceAI or Pangolin Δ scores >0.5 for splicing-impacting variants. All variants were classified according to 2015 ACMG/AMP guidelines [15]. As expected, nonsense, frameshift, and multiexonic deletions were associated with more severe phenotypes and high pathogenicity scores. In contrast, missense variants, especially those novel or affecting splicing, remain variants of uncertain significance (VUS) but are supported by conservation and predictive evidence. A detailed summary of the identified variants, their inheritance, predicted molecular consequences, and ACMG classification is provided in Table 2. Figure 5 provides an overview of the mutational landscape of the *TANGO2* gene, aiding in the interpretation of genetic alterations associated with disease.

## 3. Discussion

This Italian multicentre cohort contributes to the expanding characterisation of TANGO2-deficiency disorder (TDD), reporting two previously undescribed *TANGO2* variants and providing longitudinal clinical, neurodevelopmental, and behavioural data on five genetically confirmed patients. The findings are broadly consistent with the existing literature [4,9,19]: four out of five patients presented with metabolic crises, TANGO2 spells, or both within the first 24 months of life and exhibited developmental delay at the time of initial evaluation.

Regarding the temporal relationship between TANGO2 spells and metabolic crises, our cohort illustrates both possible sequences. Subject 2 experienced spells that typically preceded metabolic crises, while Subject 1 presented with spells without any documented metabolic decompensation. The apparent absence of spells in patients who presented initially with metabolic crisis—Subjects 4 and 5—likely reflects underrecognition rather than true absence, as these patients were very young at onset and the subtle neurological features of TANGO2 spells may not have been identified at that stage [18]. This is consistent with published reports indicating that TANGO2 spells commonly precede or accompany metabolic crises and may represent an early clinical warning sign [4,16,18]. Epilepsy is reported in approximately 40–50% of TDD individuals [4,9]; in our cohort, only Subject 4 developed epileptic seizures, symptomatic of an acute metabolic crisis rather than a chronic epileptic condition. Three out of five patients showed nonspecific interictal EEG abnormalities, and neuroimaging findings—where available—were similarly nonspecific. No early metabolic biomarkers were identified outside of acute crisis episodes, reinforcing the diagnostic challenge posed by TDD in the interictal period and the central role of genetic testing in confirming the diagnosis.

The *TANGO2* variants identified in our cohort, primarily loss-of-function mutations consistent with TDD’s recessive inheritance, demonstrate highly variable phenotypic consequences. This variability is exemplified by the divergent clinical courses observed in Subjects 2 and 3, who harbour identical compound heterozygous *TANGO2* mutations yet present with markedly different clinical phenotypes. The deletion spanning exons 3–9 represents a frequent and deleterious pathogenic allele in TDD patients. This variant has been identified as a founder effect in Latino/Hispanic and European populations, and its homozygosity is associated with substantial morbidity [2]. This mutation was also identified in Subject 1, who presented with compound heterozygosity for the p.(Ala158Val) change and exhibited mild developmental delay and no history of metabolic crises. This milder phenotype suggests that p.(Ala158Val) is a hypomorphic variant, likely retaining partial protein function, consistent with recent reports documenting the existence of milder TDD presentations—including normal or only mildly delayed development in the absence of metabolic crises—in patients carrying missense variants in compound heterozygosity with severe loss-of-function alleles [11], as observed in Subjects 1 and 3. These findings underscore the significant role of modifying factors, including genetic background, environmental influences and therapeutic interventions, in shaping phenotypic expression. The combination of previously reported and novel variants identified in our cohort further broadens the known mutational spectrum of *TANGO2*, with several variants absent from ClinVar at the time of reporting, underscoring the need for continued research to elucidate the pathogenetic relevance of newly identified mutations and to establish genotype–phenotype correlations in larger, internationally coordinated cohorts.

Intrafamilial phenotypic variability in TDD has been previously documented [4,19,20] and is further illustrated by Subjects 2 and 3. Our study adds longitudinal neurodevelopmental data and standardised adaptive and behavioural assessments to the existing description of these patients [4]. Subject 2 currently presents with moderate intellectual disability (FSIQ 42) and persistent motor impairment, while Subject 3 demonstrates age-appropriate cognitive functioning (FSIQ 93) with only mild learning difficulties. Importantly, Subject 3 had already attained all developmental milestones within normal ranges prior to diagnosis at 13 months, indicating that her favourable neurodevelopmental outcome reflects intrinsic phenotypic variability rather than an effect of early therapy. Whether genetic modifiers, sex-related factors, or random influences underlie this discordance remains unclear [19]. Preventive supplementation initiated at diagnosis may have contributed to Subject 3’s continued freedom from metabolic crises over follow-up, though this cannot be established from retrospective data alone.

A consistent observation across the surviving patients in our cohort is the absence of metabolic crises and TANGO2 spells following initiation of vitamin and cofactor supplementation. This is in line with published evidence supporting the efficacy of B-complex vitamins—particularly pantothenic acid, a coenzyme A precursor—in reducing crisis frequency and severity [4,13,15]. The underlying mechanism likely involves a combination of antioxidant effects, enhanced mitochondrial function, and support of de novo lipid biosynthesis [13,14]. However, given the retrospective and uncontrolled nature of this study and the simultaneous administration of multiple agents, causal attribution to any specific component is not possible. The dosages used across patients varied considerably, reflecting current clinical practice in the absence of standardised protocols, and are reported in Table 1 to facilitate future comparative analyses. Notably, the highest pantothenic acid doses in our cohort (500 mg/day in Subject 5) are consistent with recent reports of symptomatic improvement with high-dose B5 supplementation, though controlled prospective data remain unavailable.

A further contribution of this study is the systematic characterisation of behavioural and adaptive profiles using standardised instruments—an aspect largely unexplored in the TDD literature. Clinically significant attention problems were identified in Subject 5, with borderline scores in two additional patients. Adaptive functioning ranged from low to adequate across domains, with Communication consistently the most impaired area. These findings suggest that attentional difficulties and adaptive impairment are clinically relevant features of the TDD neurobehavioural phenotype that warrant systematic evaluation in routine clinical practice and prospective longitudinal investigation.

This study has several limitations. The cohort comprises five patients from a single country, limiting generalisability. The retrospective design precludes controlled assessment of therapeutic efficacy, and the absence of pre-treatment neurodevelopmental baselines in most patients constrains longitudinal interpretation. Despite these limitations, the comprehensive characterisation provided here, including detailed genetic annotation, neurodevelopmental trajectories, behavioural profiles and therapeutic data, adds clinically and molecularly relevant information to the growing body of evidence on TDD.

Given the phenotypic overlap of TDD with other neurometabolic and mitochondrial disorders, and the availability of potentially disease-modifying supplementation, we recommend the systematic inclusion of *TANGO2* in genetic sequencing panels for patients presenting with unexplained neurodevelopmental delay, paroxysmal neurological episodes, or recurrent rhabdomyolysis—even in the absence of overt metabolic disturbances. International collaborative registries with standardised phenotyping and genotyping protocols will be essential to accelerate the definition of genotype–phenotype correlations and the development of evidence-based therapeutic guidelines for this rare but impactful disorder.

## 4. Materials and Methods

This retrospective study enrolled five patients with genetically confirmed TDD from tertiary paediatric neurology centres across Italy. Patients were identified between June 2023 and May 2025 at the following institutions: the Ospedale dei Bambini “Pietro Barilla” (Parma), the IRCCS Arcispedale Santa Maria Nuova (Reggio Emilia), the AOU Città della Salute e della Scienza di Torino, the Department of Pediatrics at Federico II University Hospital (Napoli), and the IRCCS Ospedale Pediatrico Meyer (Firenze). Clinical data—including neurological and systemic features, laboratory findings, neuroimaging and EEG reports, and treatment outcomes—were collected through a retrospective review of medical records. The study was conducted in accordance with the Declaration of Helsinki. Written informed consent for genetic testing and use of clinical data for research purposes was obtained from the legal guardians of all participating patients prior to inclusion. Formal acknowledgment from the coordinating centre’s ethics committee (protocol no. 19357) was acquired.

### 4.1. Neurodevelopmental and Behavioural Assessment

Neurodevelopmental and behavioural assessment was performed where clinically feasible. Nonverbal cognitive ability was assessed with the Leiter International Performance Scale, Third Edition (Leiter-3) [21]. When direct testing was not feasible due to limited compliance or developmental level, adaptive functioning was assessed using the Vineland Adaptive Behaviour Scales, Second Edition (Vineland-II) [22], administered to primary caregivers. Intellectual ability was evaluated with the Wechsler Intelligence Scale for Children, Fourth Edition (WISC-IV) [23], where applicable. Behavioural profiles were assessed using the Child Behaviour Checklist (CBCL 6–18) [24], completed by parents. Where clinically indicated, additional instruments were administered, including the Conners’ Parent Rating Scale–Revised: Long Version (CPRS-R:L) [25], the Childhood Autism Rating Scale, Second Edition (CARS-2) [26], and the Short Sensory Profile [27].

### 4.2. Genetic Analysis

Exome sequencing (ES) was performed in a trio or extended familial setting. Segregation analysis was conducted by Sanger sequencing. Variant interpretation followed the 2015 guidelines of the American College of Medical Genetics (ACMG) and Genomics and the Association for Molecular Pathology (AMP) standards and guidelines [28]. Variants were annotated using the public databases ClinVar [29] and gnomAD [30], and the proprietary platforms Franklin v.92 (QIAGEN Digital Insights, Hilden, Germany) and DECIPHER v.11.38 (Wellcome Sanger Institute, Hinxton, UK) [31]. Pathogenicity of identified variants was assessed using the following in silico prediction tools: MutationTaster2021 (Charité Univesitätsmedizin Berlin, Berlin, Germany) [32], CADD v.1.7 (University of Washington, Seattle, WA, USA) [33], and phyloP100way scores obtained via the UCSC Genome Browser (University of California Santa Cruz, Santa Cruz, CA, USA) [34]. Splice site alterations were specifically evaluated using SpliceAI v.1.3.1 (Illumina Inc. San Diego, CA, USA) [35] and Pangolin (University of Toronto, Toronto, ON, Canada) [17]. No functional validation was performed; all classifications are based on computational predictions and population frequency data.

## 5. Conclusions

This case series expands the clinical and genetic understanding of TDD, highlighting its broad phenotypic heterogeneity and the life-threatening potential of metabolic crises. Our findings support the value of early diagnosis and proactive management, particularly B-vitamin and cofactor supplementation, in mitigating disease severity. Given the broad and at times subtle clinical spectrum, *TANGO2* should be included in genetic diagnostic panels for patients presenting with neurodevelopmental delay, paroxysmal neurological episodes, or unexplained rhabdomyolysis. Prospective multicentre studies and functional validation of novel variants remain essential priorities.

## Figures and Tables

**Table 1:** Clinical, neuroimaging and therapeutic features of the five patients with TANGO2-deficiency disorder. Abbreviations: GDD: global developmental delay; ID: intellectual disability; GERD: gastroesophageal reflux disease; CMPI: cow’s milk protein intolerance; CK: creatine kinase; VLCFA: very long-chain fatty acids; CSF: cerebrospinal fluid; CoQ10: coenzyme Q10. Subject 1 has been previously reported [17]; Subjects 2 and 3 have been previously reported [4].

	Subject 1	Subject 2	Subject 3	Subject 4	Subject 5
**Sex**	M	M	F	M	F
**Current age**	6 years	11 years	9 years	Deceased (13 months)	6 years
**Family history**	Negative	Sibling: Subject 3	Sibling: Subject 2	Consanguineous parents	Consanguineous parents
**Age at onset**	23 months	13 months	Asymptomatic	6 months	9 months
**GDD/ID**	Yes	Yes (ID)	No (mild learning difficulties)	Yes	Yes
**TANGO2 spells**	Yes—dystonic posturing, head tilt, emesis, irritability; precipitated by illness and reduced intake; no fixed circadian pattern	Yes—ataxia, sialorrhea, lethargy, hypotonia, hyposthenia; precipitated by illness and reduced intake	None	None	None
**Metabolic crisis**	None	Yes—rhabdomyolysis (peak CK 226,844 IU/L); cardiogenic shock (×3 episodes)	None	Yes—rhabdomyolysis, massive; fatal cardiac arrest	Yes—rhabdomyolysis (CK 34,400 IU/L), hyperammonaemia (147 µmol/L), elevated lactate, transaminases, and troponin I; QTc prolongation
**Cardiac involvement**	None	Cardiomyopathy; cardiogenic shock	None	Cardiac arrest (fatal)	QTc prolongation
**Epilepsy/EEG**	Non-epileptic spells; bitemporal background slowing, occasional sharp waves	No epilepsy; EEG unremarkable	No epilepsy; EEG unremarkable	Focal seizures during crisis; high-amplitude 2–3 Hz slow waves, prominent in occipital regions	Non-epileptic; sleep-activated posterior and frontal spikes; follow-up EEG improved
**Neuroimaging**	At follow up: bilateral temporopolar arachnoid cysts; Diffuse ventriculomegaly; enlarged CSF spaces	During acute Phase (metabolic crisis): Mild enlargement of ventricles and frontotemporal sulci; no parenchymal signal alterations. At follow-up: delayed myelination (internal capsule), thinning of the corpus callosum, and persistent frontal hyperintensities. Spectroscopy: Normal with no lactate peak.	Within normal limits	During acute Phase (metabolic crisis): Increased T2 signal in brainstem and basal ganglia increased T2 signal in the basal ganglia and thalamibilaterally, and mild global volume loss and enlarged ventricles	Not performed (sedation declined)
**Other comorbidities**	GERD	Hypothyroidism (pre-existing)	None	Poor growth; suspected CMPI and GERD	None
**Baseline laboratory**	CPK, liver enzymes, lactate, ammonia, amino acids, acylcarnitines, organic acids, VLCFA, CSF neurotransmitters: all normal	Hypothyroidism on screening	Normal	Not available	TSH and CK within normal limits at follow-up
**Chronic treatment**	CoQ10 (100 mg/day); vitamin A (400 µg/day); B-complex: B1 50 mg/day, B5 200 mg/day, B6 0.6 mg/day, B9 200 mg/day; vitamin C (25 mg/day); vitamin D3 (15 µg/day); L-carnitine (1000 mg/day); probiotics; melatonin	Levothyroxine; B-complex: B1 120 mg/day, B2 140 mg/day, B5 300 mg/day, B6 10 mg/day, B9 200 mg/day, cyanocobalamin; vitamins A, C, D; ubiquinol 100 mg/day; ubidecarenone 5 mL/day; L-carnitine 450 mg twice daily; low-fat high-carbohydrate diet	B-complex with vitamin C; pantothenic acid 150 mg/day; ubidecarenone 25 mg/day; L-carnitine 300 mg twice daily; low-fat high-carbohydrate diet	Not available	Propranolol; B-complex: thiamine 300 mg/day, riboflavin 300 mg/day, pyridoxine 150 mg/day, pantothenic acid 500 mg/day; CoQ10; vitamin C; N-acetylcysteine 240 mg/day

**Table 2:** Molecular characterisation of *TANGO2* variants reported in our cohort.

Patient	Variants (NM_152906.7)	Inheritance	CADD	phyloP100way	Splicing Prediction (SpliceAI/Pangolin)	ACMG Classification	ClinVar/Decipher
**1**	c.(179+1_180-1)_(*1?)del (exons 3-9); c.473C>T (p.Ala158Val)	Compound heterozygous (paternal deletion; maternal missense)	>24	4.2	Δ score < 0.2	LoF: Pathogenic; Missense: VUS	Deletion reported, but the specific variant is novel. Missense reported
**2**	c.262C>T (p.Arg88Ter); c.338delG (p.Gly113AlafsTer10)	Compound heterozygous (paternal nonsense; maternal frameshift)	>30	6.1	Not applicable	Both: Pathogenic	Both reported
**3**	Same as patient 2	Compound heterozygous	>30	6.1	Not applicable	Both: Pathogenic	Both reported
**4**	c.710G>A (p.Arg237Lys)	Homozygous	26.5	8.5	Δ score > 0.5 (Pangolin)	VUS	Not reported
**5**	c.188G>A (p.Gly63Asp)	Homozygous	25.3	7.8	Δ score ~0.4 (SpliceAI)	VUS	Not reported

* indicates variants identified in our patients that have been previously reported.

## Data Availability

No datasets were generated or analysed during the current study. Additional information is available from the corresponding author on reasonable request, subject to privacy restrictions.
